# Transfer RNA-derived fragment tRF-36 modulates varicose vein progression via human vascular smooth muscle cell Notch signaling

**DOI:** 10.1515/biol-2025-1075

**Published:** 2025-04-24

**Authors:** Guojun Chen, Chong Yu, Yu Shi, Danna Cai, Bin Zhou

**Affiliations:** Department of Vascular Surgery, Shanghai East Hospital Affiliated to Tongji University School of Medicine, No. 150, Jimo Road, Pudong New Area, Shanghai, 200120, P.R. China; Department of Nursing, Shanghai East Hospital Affiliated to Tongji University School of Medicine, Shanghai, 200120, P.R. China

**Keywords:** varicose veins, tRF-36, human vascular smooth muscle cells, Notch pathway, phenotypic switching

## Abstract

Varicose veins are a prevalent vascular disorder affecting millions of individuals worldwide, and we previously reported transfer RNA-derived fragment (tRF) involvement in varicose veins. This study investigated the role of tRF-36 in varicose vein pathogenesis. Varicose veins and adjacent normal vascular tissues were collected to measure the expression of Notch 1, 2, and 3 and the smooth muscle cell (SMC) markers SMA-α, and SM22α. Human vascular SMCs (HVSMCs) were transfected to alter tRF-36 levels and examine the effects on Notch 1–3, tRF-36, SMA-α, and SM22α expression. Notch 1–3 and tRF-36 levels were higher in varicose veins than in adjacent normal vascular tissues. tRF-36 knockdown decreased HVSMC viability, downregulated *Notch 1*, *2*, and *3* expression, and upregulated SMC markers (*SMA-α* and *SM22α*) compared with control HVSMCs. When the Notch pathway was inhibited, the expression of tRF-36 was significantly reduced. Additionally, Notch pathway inhibition showed similar effects to tRF-36 knockdown on HVSMC viability and the expression of *SMA-α* and *SM22α*. Furthermore, a Notch pathway inhibitor reversed the effects of the tRF-36 mimic on HVSMCs. Our study suggests a critical role for tRF-36 in varicose veins and demonstrates that tRF-36 knockdown may suppress varicose vein progression by inhibiting the Notch signaling pathway.

## 1 Introduction

Varicose veins, characterized by the dilation and tortuosity of superficial veins, are prevalent vascular disorders affecting millions of individuals worldwide [1]. This condition not only leads to aesthetic concerns but also poses significant health risks, including chronic venous insufficiency, skin ulcers, and deep vein thrombosis [2,3,4]. Treatment options for varicose veins include conservative management and surgery, depending on the clinical presentation and patient preferences [5,6,7]. Despite the availability of various treatment options, the pathogenesis of varicose veins remains unknown, and current therapies frequently do not target the underlying molecular mechanisms, resulting in high recurrence rates and limited long-term effectiveness [8]. Therefore, in-depth research into the molecular mechanisms underlying varicose veins is necessary to develop more effective treatment strategies.

In recent years, the role of non-coding RNAs in the regulation of gene expression and disease pathogenesis has gained considerable attention [9]. Transfer RNA-derived fragments (tRFs) are members of the small RNA family that have diverse biological functions, such as regulating mRNA stability, epigenetic modifications, cell proliferation, and apoptosis [10]. tRFs are reported to participate in many human diseases, such as neurodegenerative diseases [11], infectious diseases [12], and cancer [13]. Our previous study demonstrated the involvement of tRFs in varicose veins using small RNA sequencing [14]. Moreover, three differentially expressed tRFs (tRF-36 [upregulated], tRF-23 [upregulated], and tRF-40 [downregulated]) were shortlisted according to their high abundance and fold changes, and their expression levels were validated by real-time quantitative polymerase chain reaction (RT-qPCR) (*P* < 0.01 for tRF-36; *P* < 0.05 for tRF-23 and tRF-40). Based on these significant differences, we selected tRF-36 for further analysis. To the best of our knowledge, tRF-36 has only been reported in acute pancreatitis [15]. That report revealed that tRF-36 was upregulated in the serum of patients with acute pancreatitis and may act as a prospective target for acute pancreatitis. However, the exact mechanism of action of tRF-36 in varicose veins has not been elucidated.

The Notch pathway, a highly conserved intercellular communication system, plays a critical role in the decision of cell fate, tissue homeostasis, and disease progression [16,17]. In mammals, there are four Notch family receptors (Notch 1, 2, 3, and 4) and five ligands (delta-like [DLL]-1, -3, -4, Jagged-1, and -2) [18]. During angiogenesis, the Notch pathway not only regulates the proliferation and differentiation of endothelial cells, the formation of vascular branches, and the morphogenesis of blood vessels but also helps maintain the integrity of vascular endothelial cells and regulates the proliferation, migration, and contraction of vascular smooth muscle cells (VSMCs) [19]. Dysregulation of the Notch pathway has been implicated in various pathological conditions, including vascular diseases [20]. Zhao et al. [21] demonstrated that PM_2.5_ exposure could induce and aggravate atherosclerosis in rats by disrupting lipid metabolism, in which the Notch signaling pathway may play an important role. Another study showed that linarin improved restenosis after vascular injury in type 2 diabetes by modulating the disintegrin and metalloproteinase domain-containing protein 10-mediated Notch signaling pathway [22]. In addition, a recent investigation reported that in the arterial system, a transcriptional activator of endothelial Notch causes endothelial dysfunction in varicose veins through Notch4/DLL-4 signaling [23], which suggests a function of the Notch pathway in varicose veins. However, the interplay between tRF-36 and the Notch pathway in varicose veins has not yet been thoroughly investigated.

Smooth muscle cells (SMCs) play a role in vascular homeostasis in vein walls. Any alteration in the SMCs can alter the structure and function of other venous layers, resulting in increased endothelial permeability and substance release [25]. The development of varicose veins is closely related to phenotypic switching of VSMCs [24]. SMA-α and SM22α are SMC markers and are closely associated with the phenotypic switching of VSMCs, thereby playing important roles in varicose veins. Therefore, in this study, human VSMCs (HVSMCs) were used to elucidate the role of tRF-36 in varicose veins and explore its potential mechanisms of action via the Notch pathway. Understanding the molecular underpinnings of varicose veins may facilitate the development of more effective and targeted treatment strategies, ultimately improving patient outcomes and quality of life.

## 2 Materials and methods

### 2.1 Patient recruitment and sample collection

Between March and June 2021, three patients diagnosed with varicose veins were recruited at Shanghai East Hospital, Tongji University School of Medicine. Varicose vein tissues and their corresponding adjacent normal vascular tissues were collected from all patients. Collected tissues were fixed in 4% paraformaldehyde for 24 h.


**Informed consent:** Informed consent has been obtained from all individuals included in this study.
**Ethical approval:** The research related to human use has been complied with all the relevant national regulations and institutional policies and in accordance with the tenets of the Helsinki Declaration and has been approved by the Institutional Ethics Committee of Shanghai East Hospital, Tongji University School of Medicine.

### 2.2 Immunofluorescence (IF)

The fixed tissue samples were decalcified in ethylenediaminetetraacetic acid for 72 h, dehydrated with graded ethanol solutions, and embedded in paraffin. After blocks were cut into 4–5 μm thick slices, dewaxing, rehydration, antigen retrieval, and blocking with goat serum and for endogenous peroxidases were performed. Then, the tissue sections were incubated with anti-Notch 1 antibody (1:50, Notch 1# AF5307; Affinity, USA), anti-Notch 2 antibody (1:20, Notch 1# AF5296; Affinity), anti-Notch 3 antibody (1:20, Notch 1# DF7193), anti-SMA-α (1:20, SMA-α# AF1032; Affinity), or anti-SM22α (1:20, SM22α# AF9266; Affinity) at 4°C overnight. After washing, the sections were incubated with a secondary antibody at 37°C for 35 min. After washing, 4,6-diaminidine 2-phenylindole was used to stain the sections, which were then sealed with anti-fluorescence quenching sealing tablets. Images were acquired using a confocal microscope (Leica, Heidelberg, Germany).

### 2.3 Cell culture and transfection

HVSMCs were purchased from the Cell Bank, Chinese Academy of Sciences (Shanghai, China) and maintained in Dulbecco’s modified Eagle’s medium (Corning, Manassas, VA, USA; Cat # 10-013-CVR) containing 10% fetal bovine serum (Gibco, Gaithersburg, MD, USA) and 1% penicillin/streptomycin (E607011; Sangon, Shanghai, China). Then, the HVSMCs were cultured at 37°C and were passaged upon reaching 80–90% confluence.

For cell transfection, the mimics/inhibitor negative control (NC), and tRF-36 mimics/inhibitor were prepared and purchased from General Biology (Anhui, China), and cell transfection was performed as described previously [26]. Briefly, HVSMCs were seeded in 6-well plates at a density of 3 × 10^5^ cells/well. After the cell confluency reached 80–90%, the medium was changed into serum-free medium, and the HVSMCs were transfected with 5 μL mimics/inhibitor NC or tRF-36 mimics/inhibitor using Lipofectamine™ 2000 (Invitrogen, USA). After 6 h, the serum-free medium was replaced with a complete medium. Transfection efficacy was evaluated by determining the expression of tRF-36 using RT-qPCR.

### 2.4 Cell treatment

HVSMCs were seeded in 6-well plates at a density of 3 × 10^5^ cells/well. To select the optimal concentration of a kind of γ-secretase inhibitor GSI-IX (DAPT) (Notch signaling pathway inhibitor; Selleck, Houston, TX, USA; # S2215), different concentrations of DAPT (0, 2.5, 5, 10, or 20 μM) were used to treat the HVSMCs with 0 μM DAPT as the control, and the expression of Notch 1, 2, and 3 were measured using RT-qPCR. To investigate the role of tRF-36 in HVSMC growth, cells were transfected with tRF-36 mimic or inhibitor, with HVSMCs transfected with mimic NC or inhibitor NC as controls. To further explore the effects of Notch signaling pathway inhibitors, HVMSCs were divided into four groups: DMSO (control), DAPT, tRF-36 inhibitor, and DAPT + tRF-36 inhibitor.

### 2.5 Cell viability assay

The viability of HVSMCs was determined using cell counting kit-8 (CCK-8; Beyotime, Shanghai, China). HVSMCs were incubated with different treatments for 24, 48, 72, and 96 h. Then, 10 μL of CCK-8 reagent was added to cells and incubated for 2 h. The absorbance at 450 nm was determined by a microplate reader (Infinite M1000; Tecan, Switzerland).

### 2.6 RT-qPCR

Total RNA was extracted from HVSMCs after different treatments by TRIzol (Invitrogen) and reverse transcribed into cDNA with PrimeScript™ II 1st Strand cDNA synthesis kit (Takara Biomedical Technology (Beijing) Co., Ltd). The RT-qPCR reaction was carried out using SYBR green PCR master mix on an ABI Q6 system (Applied Biosystems, USA) and was initiated at 50°C for 3 min, 95°C for 3 min, followed by 40 cycles at 95°C for 10 s and 60°C for 30 s. The relative expression levels of *tRF-36*, *Notch 1*, *2*, *3*, and *4*, *SMA-α*, and *SM22α* were calculated using the 2^−△△Ct^ method. The primers are listed in Table 1.

**Table 1:** The sequences of all primers used in this study

Primers	Primer sequence (5′–3′)
Actin-F	AGCACAGAGCCTCGCCTTTG
Actin-R	CTTCTGACCCATGCCCACCA
U6-F	CGATACAGAGAAGATTAGCATGGC
U6-R	AACGCTTCACGAATTTGCGT
Notch1-F	CTCATCAACTCACACGCCGA
Notch1-R	GGTGTCTCCTCCCTGTTGTTCT
Notch2-F	GTATTGGCTCCCTGTTCCCC
Notch2-R	AATGGTACACCGCTGACCTTG
Notch3-F	AGGTGATCGGCTCGGTAGTAAT
Notch3-R	CTGACAACGCTCCCAGGTAGT
Notch4-F	GCCCCTCCCACTCTCGGT
Notch4-R	TCACAGTCGTAGCCATCAAACAG
SMAα-F	CCAGCTATGTGTGAAGAAGAGGAC
SMAα-R	CTTTTTGTCCCATTCCCACCA
SM22α-F	AATGGCGTGATTCTGAGCAAG
SM22α-R	CCATAGTCCTCAGCCGCCT
tRF-36-RT	GTCGTATCCAGTGCGTGTCGTGGAGTCGG
	CAATTGCACTGGATACGACGGGTGAA
tRF-36-F	ACATGGTCTAGCGGTTAGGATTC
Downstream universal primer	AGTGCGTGTCGTGGAGTCG

### 2.7 Western blot

Total protein was isolated from HVSMCs after different treatments and quantified using a BCA protein assay kit (Boster Biological Technology Co., Ltd, Wuhan, China). Subsequently, the total protein (20 μg) was separated and transferred onto polyvinylidene fluoride membranes. After blocking with 5% skim milk, the membranes were incubated with the anti-Notch 1 antibody (1:1,000), anti-Notch 2 antibody (1:1,000), anti-Notch 3 antibody (1:500), or anti-GAPDH antibody (1:5,000) at 4°C overnight. Afterward, the membranes were incubated with an HRP-conjugated secondary antibody (1:1,000; Beyotime) at 37°C for 2 h. After washing, the bands were visualized using an enhanced chemiluminescence assay kit (Thermo).

### 2.8 Statistical analysis

Data are shown as mean ± standard deviation. Data were compared using Student’s *t*-test (for two groups) or one-way analysis of variance followed by Dunnett’s multiple comparison post hoc tests (for more than two groups) using GraphPad Prism software. Statistical significance was set at *P* < 0.05.

## 3 Results

### 3.1 Expression of Notch 1, 2, and 3 in varicose vein tissues

**Figure 1:**
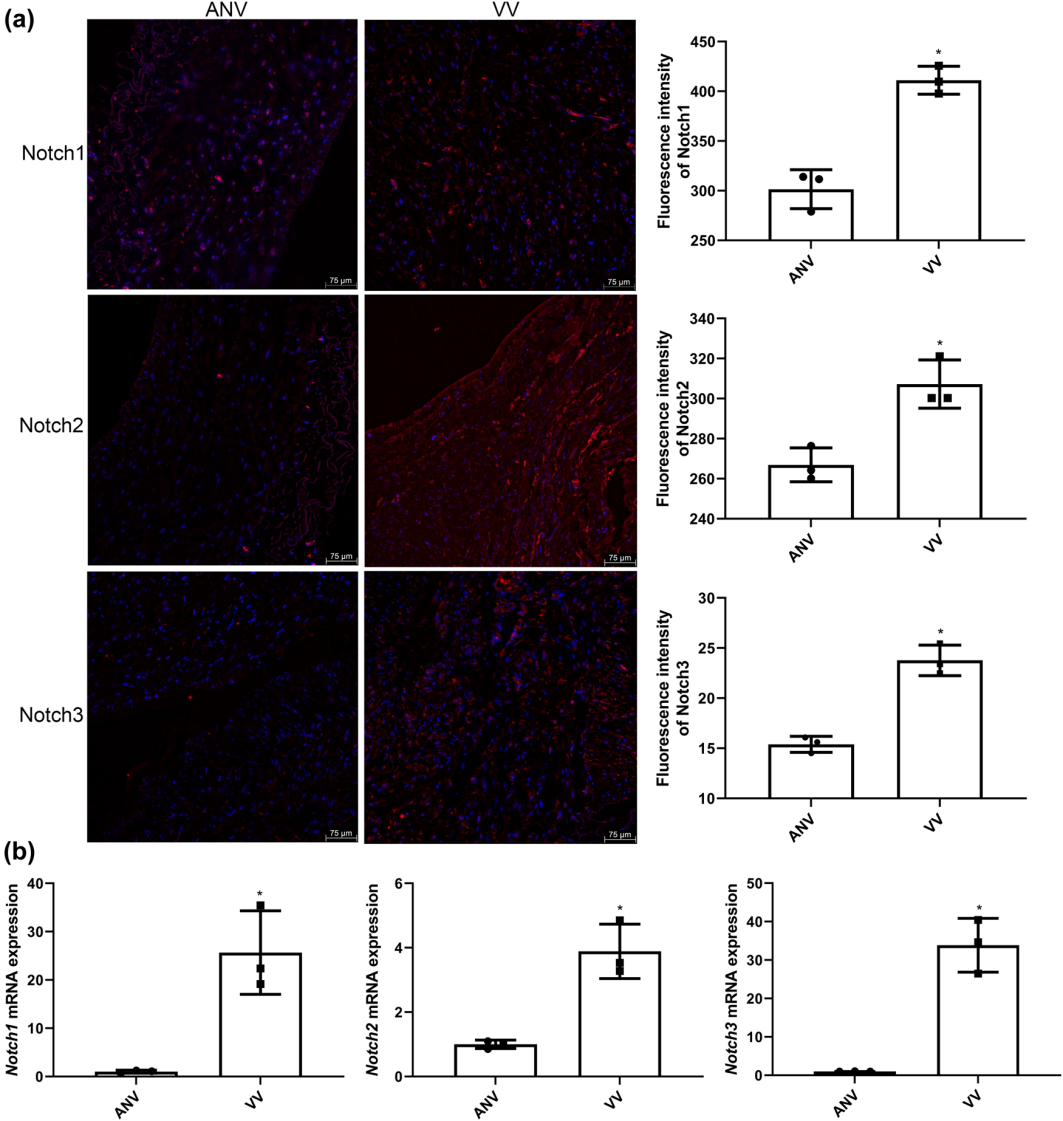
The expression of Notch 1, 2, and 3 in varicose vein (VV) tissues and adjacent normal vein (ANV) samples. (a) The fluorescence intensity of Notch 1, 2, and 3 detected by IF. (b) The mRNA expression of *Notch 1*, *2*, and *3* measured by real-time quantitative PCR (RT-qPCR). *P < 0.05 compared to ANV.

Varicose veins and adjacent normal vascular tissues were collected from participants to detect the expression of Notch 1, 2, and 3 by IF and RT-qPCR. IF showed that Notch 1 fluorescence intensity was significantly elevated in varicose vein tissues compared to adjacent normal tissues (*P* < 0.05); similar results were also observed for Notch 2 and 3 expressions (Figure 1a). In addition, the trend of *Notch1/2/3* mRNA expression in varicose vein tissues and adjacent normal vascular tissues determined by RT-qPCR was consistent with that of Notch 1/2/3 fluorescence intensity detected by IF (Figure 1b). These results indicate that Notch 1/2/3 could be expressed at higher levels in varicose veins.

### 3.2 Expression of SMA-α and SM22α in varicose vein tissues

**Figure 2:**
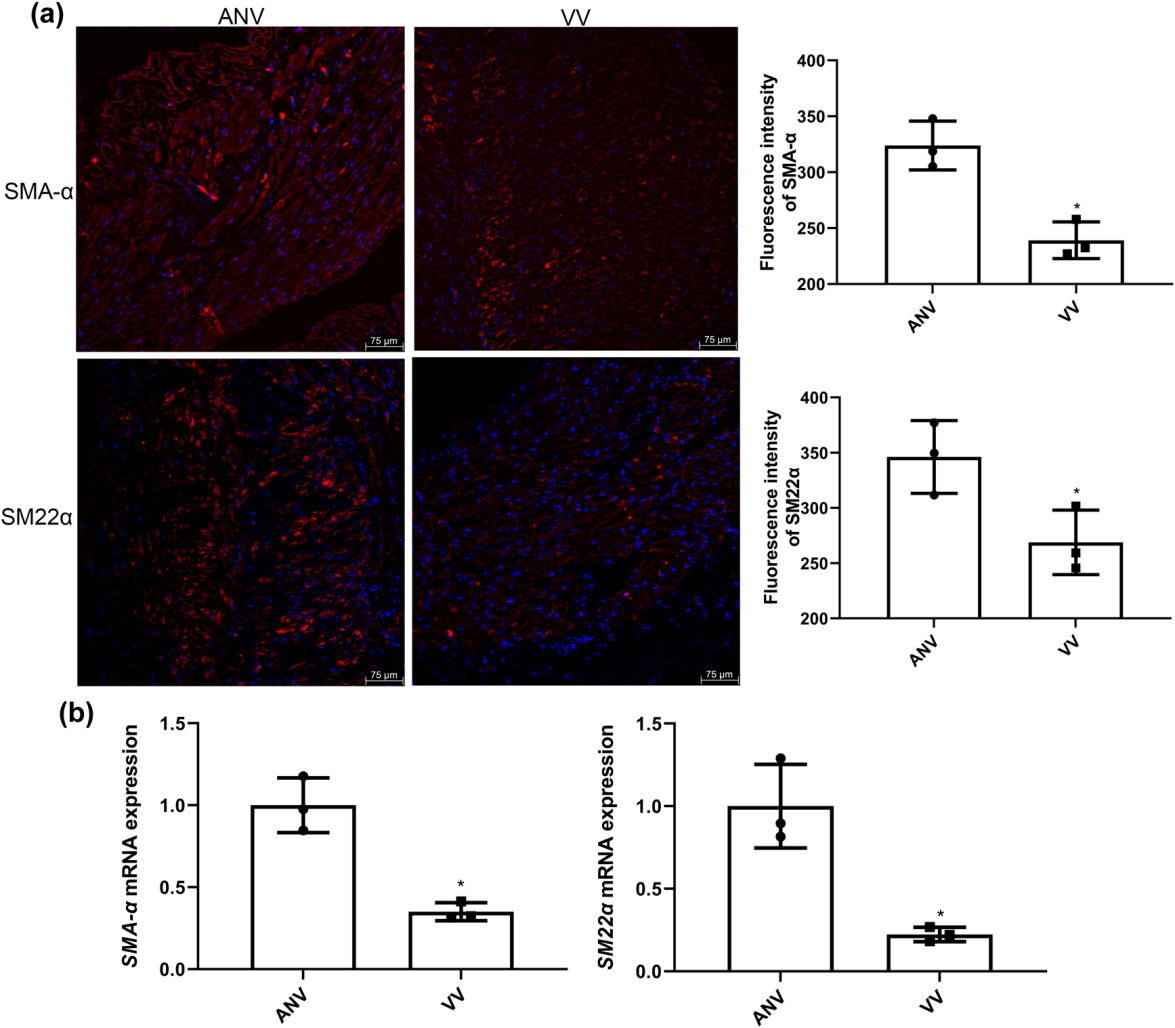
Expression of SMA-α and SM22α in varicose vein tissues. (a) The fluorescence intensity of SMA-α and SM22α detected by IF. (b) The mRNA expression of *SMA-α* and *SM22α* measured by RT-qPCR. *P < 0.05 compared to ANV.

To further explore the varicose vein phenotype in human tissue samples, the expression of smooth muscle markers (SMA-α and SM22α) in varicose vein tissues and adjacent normal vascular tissues was measured by IF and RT-qPCR. The fluorescence intensity of SMA-α and SM22α was significantly reduced in the varicose vein tissues in comparison with the adjacent normal vascular tissues (*P* < 0.05; Figure 2a). Furthermore, RT-qPCR demonstrated similar reductions in the SMA-α and SM22α expression in varicose vein tissues compared with adjacent normal vasculature (Figure 2b).

### 3.3 Cell transfection efficiency and selection of the optimal DAPT concentration

**Figure 3:**
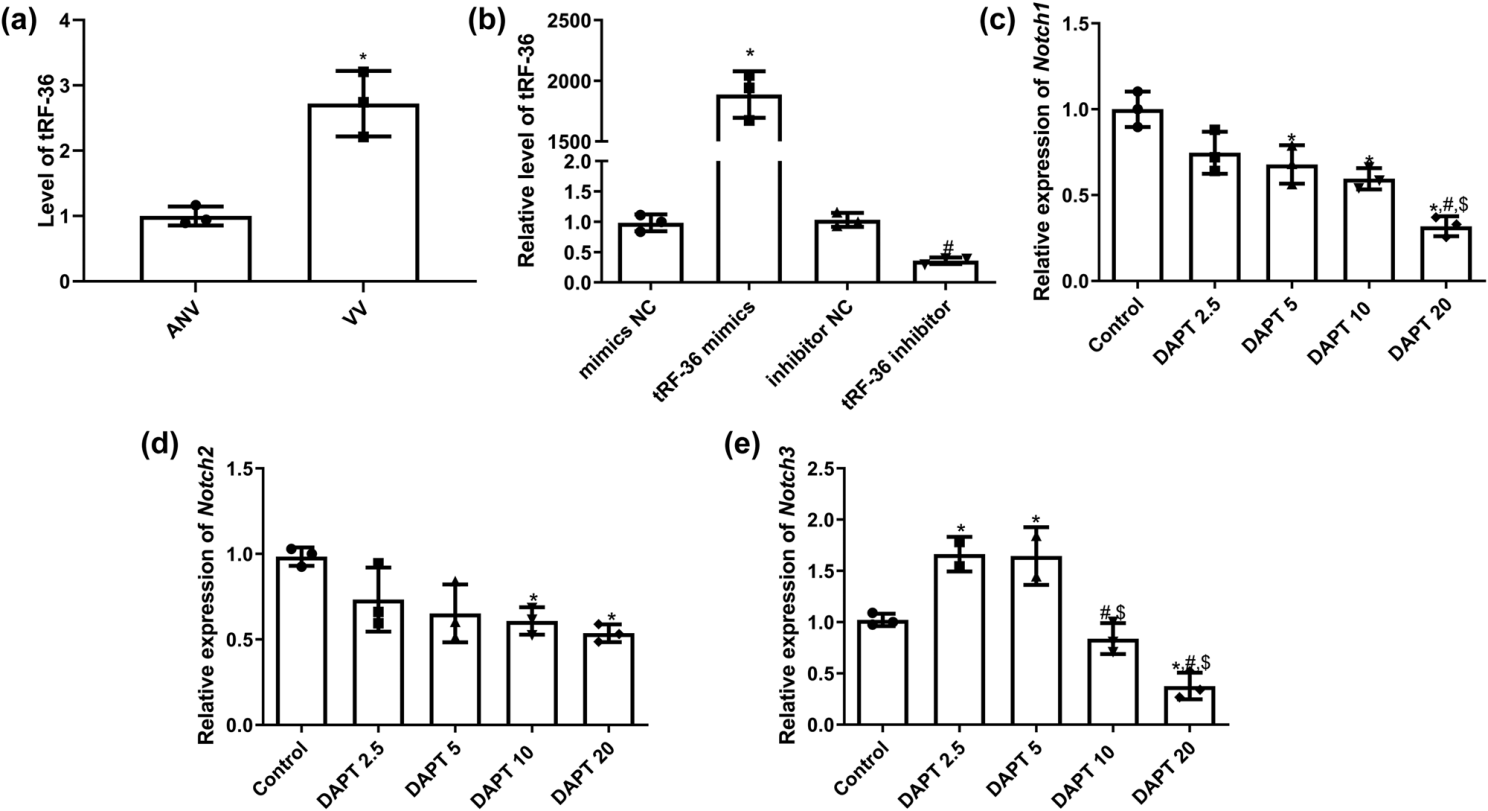
Cell transfection efficiency and selection of the optimal DAPT concentration. (a) The level of *tRF-36* in the varicose vein tissues measured by RT-qPCR. *P < 0.05 compared to ANV. (b) The expression level of tRF-36 after cell transfection detected by RT-qPCR. *P < 0.05 compared to mimic NC and ^#^ *P* < 0.05 compared to inhibitor NC. The expression of Notch 1 (c), 2 (d), and 3 (e) after cells treated with different concentrations of DAPT. *P < 0.05 compared to control, ^#^ *P* < 0.05 compared to DAPT 2.5, and ^$^ *P* < 0.05 compared to DAPT 5.

Compared with adjacent normal vascular tissues, the level of tRF-36 was significantly higher in varicose vein tissues (*P* < 0.05; Figure 3a). Therefore, to study the effects of tRF-36 on the growth of HVSMCs, HVSMCs with tRF-36 overexpression and knockdown were established, and their transfection efficiency was confirmed by RT-qPCR. There was no significant difference in tRF-36 expression between the mimic NC and inhibitor NC groups (*P* > 0.05), and tRF-36 expression increased significantly after transfection with tRF-36 mimics (*P* < 0.05) but was decreased after transfection with the tRF-36 inhibitor (*P* < 0.05; Figure 3b). These results suggested that HVSMCs with tRF-36 overexpression and knockdown were successfully established.

To determine the optimal concentration of DAPT, HVSMCs were treated with different concentrations of DAPT, and the expression of Notch 1, 2, and 3 was measured. When the DAPT concentration was 20 μM, the expressions of Notch 1, 2, and 3 were significantly downregulated compared to the control cells (*P* < 0.05; Figure 3c–e) and showed an optimal inhibition effect on the Notch signaling pathway. Therefore, we selected 20 μM DAPT to treat HVSMCs in the subsequent experiments.

### 3.4 Effects of tRF-36 on the growth of HVSMCs

**Figure 4:**
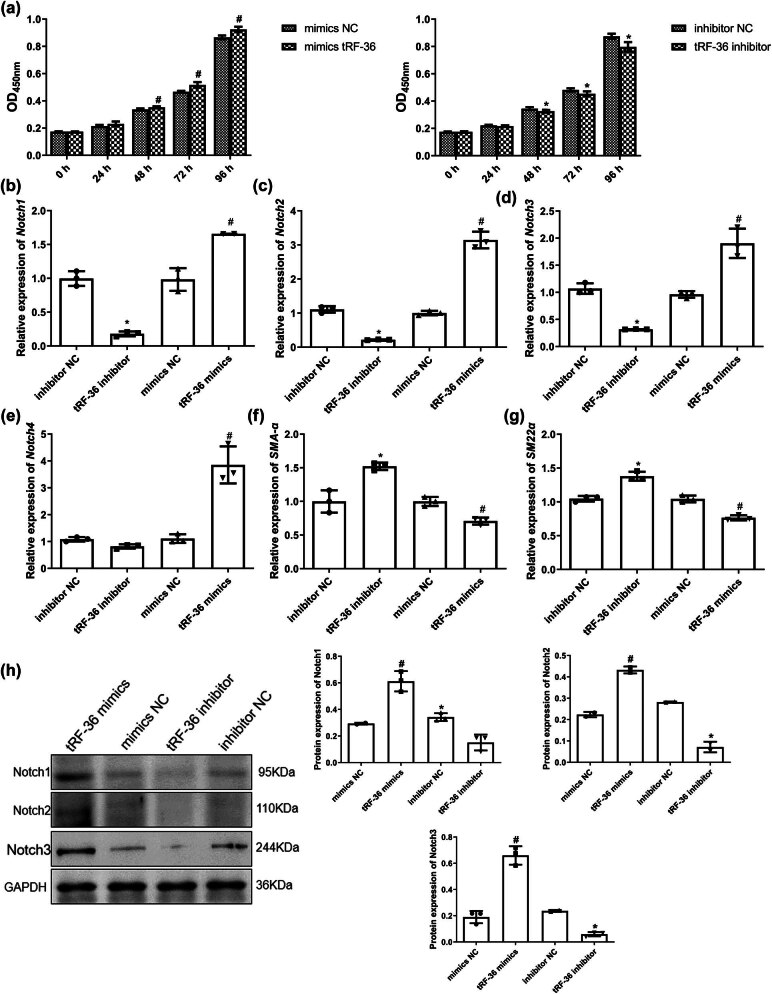
Effects of tRF-36 on the growth of HVSMCs. (a) The cell viability in the HVSMCs after transfection with tRF-36 inhibitor or tRF-36 mimic detected by CCK-8. The mRNA expression levels of *Notch 1* (b), *2* (c), *3* (d), and *4* (e) after cell transfection detected by RT-qPCR. The mRNA levels of *SMA-α* (f) and *SM22α* (g) after cell transfection detected by RT-qPCR. (h) The protein levels of Notch 1, 2, and 3 after cell transfection detected by western blot. *P < 0.05 compared to inhibitor NC and ^#^ *P* < 0.05 compared to mimics NC.

Subsequently, we examined the effects of tRF-36 on HVSMC growth. There was no significant difference in HVSMC viability between the inhibitor NC and tRF-36 inhibitor groups or the mimic NC and tRF-36 mimic groups after 24 h of culture (*P* > 0.05; Figure 4a). However, after culturing for 48, 72, and 96 h, the viability of HVSMCs was significantly inhibited in the tRF-36-knockdown HVSMCs compared to the cells transfected with inhibitor NC (*P* < 0.05), whereas viability was markedly enhanced in the tRF-36-overexpressed HVSMCs in comparison to the NC group (*P* < 0.05; Figure 4a). Next, the expression levels of Notch pathway-related genes (*Notch 1*, *2*, *3*, and *4*) were measured. Compared to the corresponding NC group, the mRNA expression of *Notch 1*, *2*, and *3* was significantly upregulated after tRF-36 overexpression (*P* < 0.05), whereas they were markedly downregulated after tRF-36 knockdown (*P* < 0.05; Figure 4b–d). *Notch 4* mRNA expression was not significantly changed after transfection with the tRF-36 inhibitor (*P* > 0.05) but was upregulated after transfection with tRF-36 mimics (*P* < 0.05; Figure 4e). Smooth muscle marker (*SMA-α* and *SM22α*) expression was significantly higher in the HVSMCs with tRF-36 knockdown than that in the inhibitor NC group (*P* < 0.05) but was lower in the HVSMCs with tRF-36 overexpression than that in the mimics NC group (*P* < 0.05; Figure 4f and g). In addition, the tendencies of Notch 1, 2, and 3 protein levels were consistent with their mRNA levels (*P* < 0.05; Figure 4h).

### 3.5 Effects of the Notch pathway on the growth of HVSMCs

**Figure 5:**
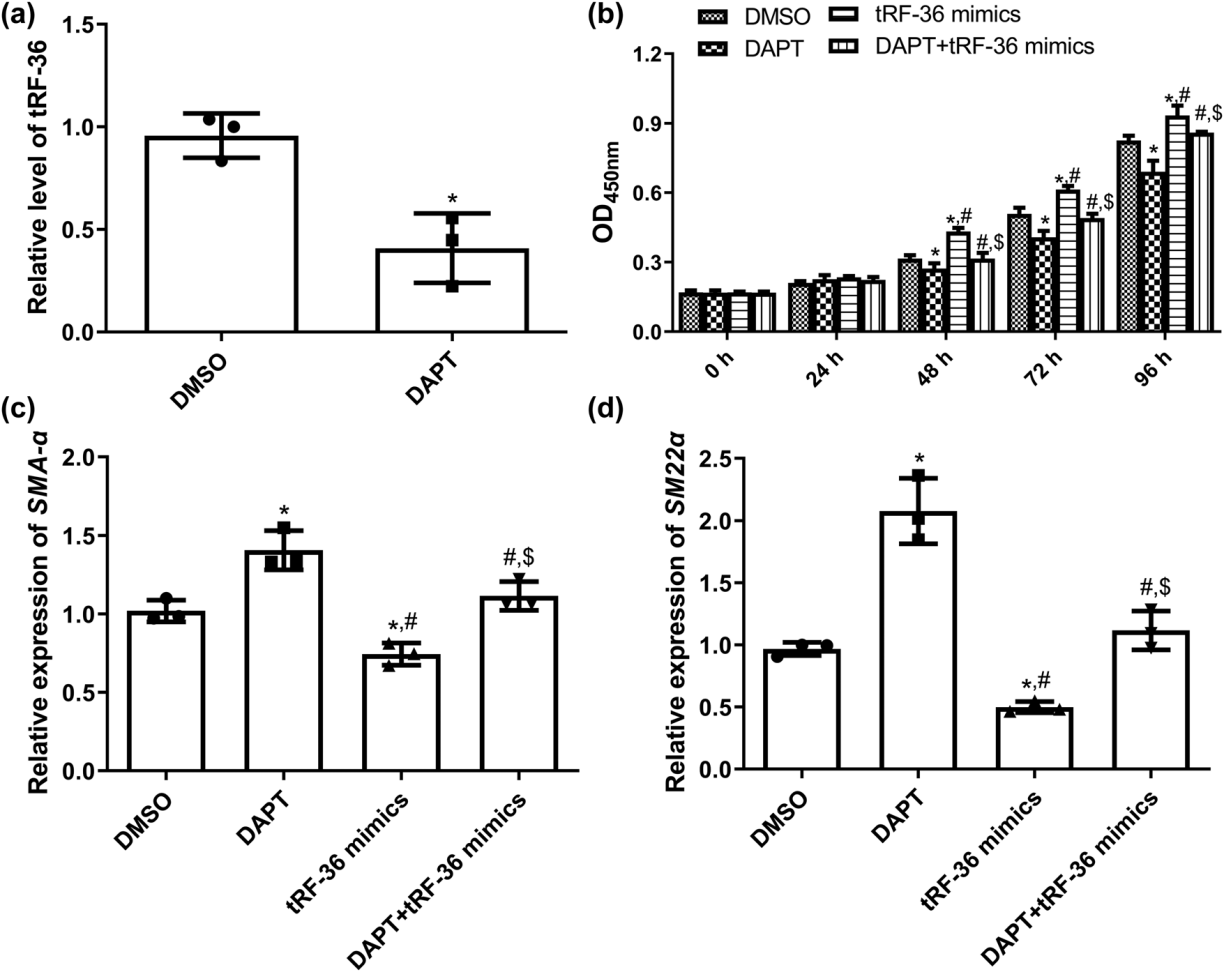
Effects of Notch pathway on the growth of HVSMCs. (a) The mRNA expression of *tRF-36* after cells treated with DAPT detected by RT-qPCR. (b) The viability of HVSMCs after cell transfection or DAPT treatment detected by CCK-8. The mRNA expression of *SMA-α* (c) and *SM22α* (d) after cell transfection or DAPT treatment detected by RT-qPCR. *P < 0.05 compared to the DMSO group, ^#^ *P* < 0.05 compared to the DAPT group, and ^$^ *P* < 0.05 compared to the tRF-36 mimics group.

To further investigate whether tRF-36 regulates varicose vein progression through the Notch pathway, we inhibited the Notch pathway with DAPT and detected the expression of tRF-36. As expected, after HVSMCs were treated with DAPT, the expression of tRF-36 was significantly reduced compared to that in cells treated with DMSO (*P* < 0.05; Figure 5a). After 48, 72, and 96 h of culture, the viability of HVSMCs was significantly reduced after DAPT treatment compared to the DMSO group (*P* < 0.05), while viability was increased after transfection with tRF-36 mimics (*P* < 0.05). In the DAPT + tRF-36 mimics group, the viability was restored to a level similar to that in the DMSO group (*P* > 0.05; Figure 5b). Furthermore, the mRNA expression of *SMA-α* and *SM22α* was significantly upregulated after DAPT treatment compared with the DMSO group (*P* < 0.05), while they were downregulated after transfected with tRF-36 mimics (*P* < 0.05; Figure 5c and d). No significant difference in the *SMA-α* and *SM22α* mRNA expression was observed between the DMSO and DAPT + tRF-36 mimics groups (*P* > 0.05; Figure 5c and d). These outcomes implied that DAPT reversed the effects of tRF-36 mimics on the viability of HVSMCs and the expression of *SMA-α* and *SM22α*.

## 4 Discussion

Varicose veins can damage blood vessels, cause painful swelling, lead to thrombosis, increase in prevalence with age, and affect a person’s productivity and quality of life [27,28]. The global prevalence of varicose veins remains high, occurring in 1–73% of women and 2–56% of men [29,30]. However, the pathogenesis of varicose veins remains unclear and the pathogenic mechanisms remain elusive preventing the development of new treatments [31]. tRF-36 is upregulated in the vascular tissue of patients with varicose veins [14] and the Notch signaling pathway has been implicated in varicose veins [23]. However, the interaction between tRF-36 and the Notch pathway in varicose veins had not previously been explored. Here, we found that the Notch pathway was potentially activated in varicose veins and that tRF-36 levels were significantly higher in varicose vein tissues than in adjacent normal vascular tissues. Compared with the NC groups, tRF-36 knockdown significantly decreased the viability of HVSMCs, downregulated the expression of Notch 1, 2, and 3, and upregulated smooth muscle markers (*SMA-α* and *SM22α*). In addition, DAPT (a Notch pathway inhibitor) had similar effects to tRF-36 knockdown and could reverse the effects of tRF-36 mimics on the viability of HVSMCs and the expression of *SMA-α* and *SM22α*. Therefore, we speculated that tRF-36 knockdown may suppress varicose vein progression by inhibiting the Notch signaling pathway.

Varicose veins are associated with abnormal dilation and dysfunction of blood vessel wall [29]. HVSMCs play a central role in the normal functioning of blood vessels, including vasoconstriction, vessel wall maintenance, and repair [32]. Additionally, abnormal behavior of SMCs, such as excessive proliferation, migration, or apoptosis, may be related to the development of varicose veins [33,34]. Phenotypic abnormalities of SMCs in varicose veins have been reported to cause functional disturbance [34]. Therefore, in this study, we used HVSMCs to investigate the function of tRF-36 in varicose veins.

Although the role of tRFs in many biological processes has been extensively investigated, their role in varicose veins has not been fully explored [35]. A previous study suggested that tRFs, including tRF-36, tRF-23, and tRF-40, are differentially expressed in varicose veins [14]. Recently, Mahmutoglu et al. [36] investigated the correlation between tRF-36, tRF-23, and tRF-40 and varicocele progression. The results showed that tRF-36 may play a role in varicocele progression; however, the exact mechanism remained unclear. This study showed that tRF-36 was highly expressed in the varicose veins, and tRF-36 knockdown significantly decreased the viability of HVSMCs and increased the expression of *SMA-α* and *SM22α*. As non-terminally differentiated cells, VSMCs have two characteristic phenotypes: contractile and synthetic [37]. The VSMCs in adults are mainly of the contractile phenotype, and the SM22α is highly expressed in the cytoplasm. When the intima of the vascular wall is damaged or the vascular wall is stimulated by various factors, the VSMCs transform from a contractile phenotype into a synthetic one [38]. SMA-α is a vascular smooth muscle actin, which is mainly synthesized and expressed in contractile phenotype VSMCs [39]. Chen et al. [40] reported that SM22α and SMA-α were downregulated in varicose veins, indicating that VSMCs had transformed the contractile phenotype into a synthetic phenotype. Phenotypic changes in VSMCs promote venous wall remodeling, leading to the development of varicose veins. Taken together, it can be inferred that tRF-36 knockdown could upregulate the expression of α-SMA and SM22α. Thus, tRF-36 knockdown may be conducive to the transformation of VSMCs into a contractile phenotype, indicating the significance of tRF-36 in veins.

The Notch pathway is involved in many biological processes, including cell proliferation, fate determination, differentiation, and apoptosis. In the vascular system, the Notch pathway is critical for normal development and functional maintenance of blood vessels [41]. Notch signaling regulates the proliferation and migration of VSMCs, which play a critical role in the development of varicose veins [42]. Aberrant Notch signaling may lead to excessive proliferation and migration of VSMCs, resulting in abnormal dilation of the vessel wall [43]. In the present study, Notch 1, 2, and 3 were upregulated in varicose vein tissue samples, as detected by IF, suggesting that the Notch signaling pathway is potentially activated in varicose veins. The expression of tRF-36 was significantly decreased when the Notch pathway was inhibited. Additionally, inhibition of the Notch pathway resulted in similar effects as tRF-36 knockdown on the viability of HVSMCs and the expression of *SMA-α* and *SM22α*. Further studies revealed that Notch pathway inhibition reversed the effects of the tRF-36 mimic on the viability of HVSMCs. A previous study by Sreelakshmi et al. [23] demonstrated that Notch components are highly expressed in the neointima of varicose veins, and in vitro experiments have shown that even small changes in the venous flow pattern can enhance the ETS1/Notch signaling pathway, indicating that the Notch pathway plays an important role in the progression of varicose veins. Another study showed that forkhead box C2 (FOXC2) and FOXC2 antisense RNA 1 (FOXC2-AS1) were upregulated in varicose veins, and the overexpression of FOXC2-AS1 promoted phenotypic transition, proliferation, and migration of human great saphenous vein SMCs by activating the Notch pathway. However, both FOXC2 silencing and the Notch signaling inhibitor FLI-06 could eliminate the effects of FOXC2-AS1 overexpression [44]. Taken together with our results, these reports suggest that tRF-36 knockdown may inhibit the growth and phenotypic transition of HVSMCs by inhibiting the Notch pathway, thereby suppressing varicose vein development. However, it should be noted that the specific roles and mechanisms of Notch signaling in varicose veins are quite complex and require further exploration.

This study has some limitations. First, the sample size of three patients was small, which limits the generalizability of the findings. Therefore, our conclusions need to be further verified in a larger cohort. Second, the limitations of the assays used, such as the potential cross-reactivity of antibodies in IF and the efficiency and specificity of transfection reagents, should be considered in the future. In addition, our results must be verified through *in vivo* experiments.

**Figure 6:**
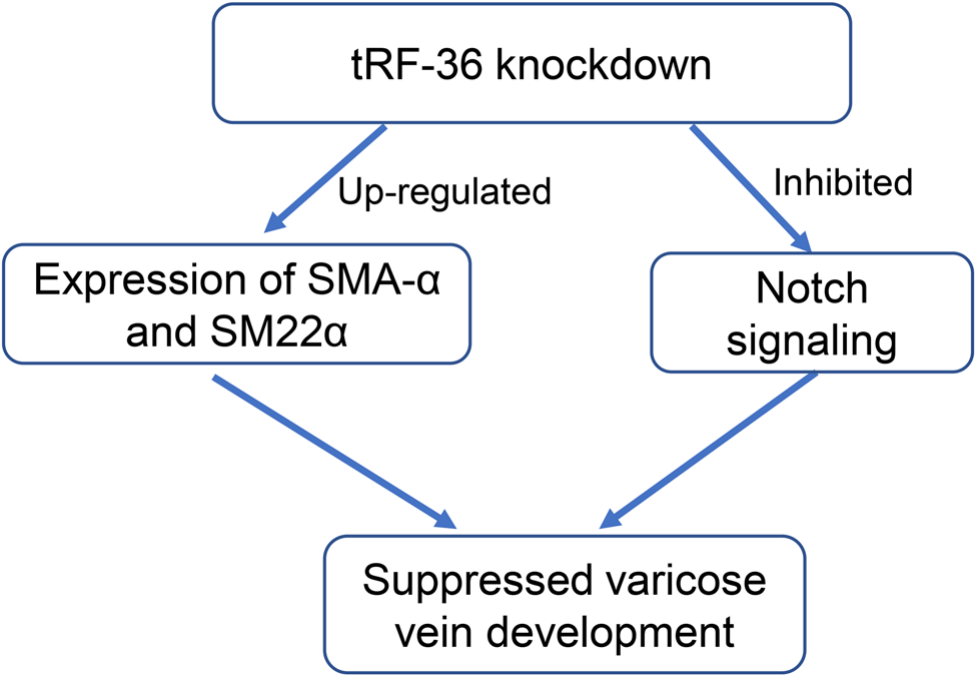
The relationships between varicose vein, tRF-36, and Notch signaling.

In conclusion, our study suggested the critical role of tRF-36 in varicose veins. Importantly, we demonstrated for the first time that tRF-36 knockdown suppressed varicose vein development by inhibiting the Notch pathway (Figure 6). Our study provides new evidence for the functional roles of tRF-36 in varicose veins and lays the foundation for the development of effective therapeutic strategies based on the knockdown of tRF-36 and inhibition of the Notch pathway in varicose veins.

## References

[1] Wang M, Sharma AK. Varicose veins. J Radiol Nurs. 2019;38:150–4.

[2] Angle N, Bergan JJ. Varicose veins: chronic venous insufficiency. Vascular surgery: A comprehensive review, 5th edn. Philadelphia, PA: Saunders; 1998. p. 800–7.

[3] Alsaigh T, Fukaya E. Varicose veins and chronic venous disease. Cardiol Clin. 2021;39:567–81. 10.1016/j.ccl.2021.06.009 34686268

[4] Raetz J, Wilson M, Collins K. Varicose veins: diagnosis and treatment. Am Fam Physician. 2019;99:682–8. 31150188

[5] Li X, Yang B, Li X, Ren S. Prospective comparison of effect of ligation and foam sclerotherapy with foam sclerotherapy alone for varicose veins. Ann Vasc Surg. 2018;49:75–9. 10.1016/j.avsg.2018.01.004 29428536

[6] Li X, Ren S, Li X. Outcomes of foam sclerotherapy plus ligation versus foam sclerotherapy alone for venous ulcers in lower extremities. Ann Vasc Surg. 2017;45:160–5. 10.1016/j.avsg.2017.06.055 28648655

[7] Gao R-D, Qian S-Y, Wang H-H, Liu Y-S, Ren S-Y. Strategies and challenges in treatment of varicose veins and venous insufficiency. World J Clin Cases. 2022;10:5946. 10.12998/wjcc.v10.i18.5946 PMC9254182 35949828

[8] Dwerryhouse S, Davies B, Harradine K, Earnshaw JJ. Stripping the long saphenous vein reduces the rate of reoperation for recurrent varicose veins: five-year results of a randomized trial. J Vasc Surg. 1999;29:589–92. 10.1016/s0741-5214(99)70302-2 10194484

[9] Leeper NJ, Maegdefessel L. Non-coding RNAs: key regulators of smooth muscle cell fate in vascular disease. Cardiovasc Res. 2018;114:611–21. 10.1093/cvr/cvx249 PMC5852528 29300828

[10] Zhao Y, Wang K, Zhao C, Liu N, Wang Z, Yang W, et al. The function of tRNA-derived small RNAs in cardiovascular diseases. Mol Therapy-Nucleic Acids. 2024;35(1):102114. 10.1016/j.omtn.2024.102114 PMC10835008 38314096

[11] Ivanov P, Emara MM, Villen J, Gygi SP, Anderson P. Angiogenin-induced tRNA fragments inhibit translation initiation. Mol Cell. 2011;43:613–23. 10.1016/j.molcel.2011.06.022 PMC3160621 21855800

[12] Garcia-Silva MR, Cabrera-Cabrera F, Güida MC, Cayota A. Novel aspects of tRNA-derived small RNAs with potential impact in infectious diseases. Adv Biosci Biotechnol. 2013;4(5A):17–25.

[13] Pekarsky Y, Balatti V, Palamarchuk A, Rizzotto L, Veneziano D, Nigita G, et al. Dysregulation of a family of short noncoding RNAs, tsRNAs, in human cancer. Proc Natl Acad Sci. 2016;113:5071–6. 10.1073/pnas.1604266113 PMC4983805 27071132

[14] Yu C, Wang X, Hong Y, Chen G, Ge J, Cao H, et al. Expression profile of tRNA‑derived fragments and their potential roles in human varicose veins. Mol Med Rep. 2019;20:3191–201. 10.3892/mmr.2019.10544 PMC6755252 31432124

[15] Fan X-R, Huang Y, Su Y, Chen S-J, Zhang Y-L, Huang W-K, et al. Exploring the regulatory mechanism of tRNA-derived fragments 36 in acute pancreatitis based on small RNA sequencing and experiments. World J Gastroenterol. 2023;29:4642. 10.3748/wjg.v29.i30.4642 PMC10472903 37662862

[16] Artavanis-Tsakonas S, Rand MD, Lake RJ. Notch signaling: cell fate control and signal integration in development. Science. 1999;284:770–6. 10.1126/science.284.5415.770 10221902

[17] Gozlan O, Sprinzak D. Notch signaling in development and homeostasis. Development. 2023;150:dev201138. 10.1242/dev.201138 36794955

[18] Gridley T. Notch signaling in the vasculature. Curr Top Dev Biol. 2010;92:277–309. 10.1016/S0070-2153(10)92009-7 PMC3659771 20816399

[19] Kukita K, Matsuzaka N, Takai M, Imamura Y, Shin Y. Notch signaling pathway induces expression of type IV collagen in angiogenesis. J Biochem. 2024;175:539–49. 10.1093/jb/mvad120 38167713

[20] Iso T, Hamamori Y, Kedes L. Notch signaling in vascular development. Arterioscler Thrombo Vasc Biol. 2003;23:543–53. 10.1161/01.ATV.0000060892.81529.8F 12615665

[21] Zhao T, Li X, Qian H, Miao X, Zhu Y, Wang J, et al. PM(2.5) induces the abnormal lipid metabolism and leads to atherosclerosis via Notch signaling pathway in rats. Toxicology. 2023;485:153415. 10.1016/j.tox.2022.153415 36603807

[22] Jiang A, Liu L, Wang J, Liu Y, Deng S, Jiang T. Linarin ameliorates restenosis after vascular injury in type 2 diabetes mellitus via regulating ADAM10-mediated notch signaling pathway. Cardiovasc Toxicol. 2024;24:587–97. 10.1007/s12012-024-09863-4 38691303

[23] Sreelakshmi BJ, Karthika CL, Ahalya S, Kalpana SR, Kartha CC, Sumi S. Mechanoresponsive ETS1 causes endothelial dysfunction and arterialization in varicose veins via NOTCH4/DLL4 signaling. Eur J Cell Biol. 2024;103:151420. 10.1016/j.ejcb.2024.151420 38759515

[24] Xiao Y, Huang Z, Yin H, Lin Y, Wang S. In vitro differences between smooth muscle cells derived from varicose veins and normal veins. J Vasc Surg. 2009;50:1149–54. 10.1016/j.jvs.2009.06.048 19703751

[25] Ortega MA, Romero B, Asúnsolo Á, Sainz F, Martinez-Vivero C, Álvarez-Mon M, et al. Behavior of smooth muscle cells under hypoxic conditions: possible implications on the varicose vein endothelium. BioMed Res Int. 2018;2018:7156150. 10.1155/2018/7156150 PMC6220744 30498761

[26] Zhou Q, Yang H, Pan H, Pan H, Zhou J. Exosomes isolated from the miR-215-modified bone marrow mesenchymal stem cells protect H2O2-induced rat myoblasts via the miR-215/FABP3 pathway. Exp Mol Pathol. 2021;119:104608. 10.1016/j.yexmp.2021.104608 33503452

[27] Whiteley MS. Current best practice in the management of varicose veins. Clin Cosmet Invest Dermatol. 2022;15:567–83. 10.2147/CCID.S294990 PMC8995160 35418769

[28] Gawas M, Bains A, Janghu S, Kamat P, Chawla P. A comprehensive review on varicose veins: preventive measures and different treatments. J Am Nutr Assoc. 2022;41:499–510. 10.1080/07315724.2021.1909510 34242131

[29] Raffetto J, Khalil R. Mechanisms of varicose vein formation: valve dysfunction and wall dilation. Phlebology. 2008;23:85–98. 10.1258/phleb.2007.007027 18453484

[30] Beebe-Dimmer JL, Pfeifer JR, Engle JS, Schottenfeld D. The epidemiology of chronic venous insufficiency and varicose veins. Ann Epidemiol. 2005;15:175–84. 10.1016/j.annepidem.2004.05.015 15723761

[31] Chandran Latha K, Sreekumar A, Beena V, SS BR, Lakkappa RB, Kalyani R, et al. Shear stress alterations activate BMP4/pSMAD5 signaling and induce endothelial mesenchymal transition in varicose veins. Cells. 2021;10:3563. 10.3390/cells10123563 PMC8700678 34944071

[32] Bacakova L, Travnickova M, Filova E, Matějka R, Stepanovska J, Musilkova J, et al. The role of vascular smooth muscle cells in the physiology and pathophysiology of blood vessels. Muscle Cell Tissue-Current Status Res Field. 2018;1:13.

[33] Urbanek T, Skop B, Wiaderkiewicz R, Wilczok T, Ziaja K, Lebda-Wyborny T, et al. Smooth muscle cell apoptosis in primary varicose veins. Eur J Vasc Endovasc Surg. 2004;28:600–11. 10.1016/j.ejvs.2004.09.008 15531194

[34] Xu Y, Bei Y, Li Y, Chu H. Phenotypic and functional transformation in smooth muscle cells derived from varicose veins. J Vasc Surg: Venous Lymphatic Disord. 2017;5:723–33. 10.1016/j.jvsv.2017.04.009 28818228

[35] Weng Q, Wang Y, Xie Y, Yu X, Zhang S, Ge J, et al. Extracellular vesicles-associated tRNA-derived fragments (tRFs): biogenesis, biological functions, and their role as potential biomarkers in human diseases. J Mol Med. 2022;100:679–95. 10.1007/s00109-022-02189-0 PMC9110440 35322869

[36] Mahmutoglu AM, Caniklioglu M, Caniklioglu A, Kaymak E, Terzi Y. Association between transfer RNA-derived fragments and varicocele. Fertil Steril. 2023;120:e274.

[37] Wen J-kHan, M, Zheng B, Yang S-L. Comparison of gene expression patterns and migration capability at quiescent and proliferating vascular smooth muscle cells stimulated by cytokines. Life Sci. 2002;70:799–807. 10.1016/s0024-3205(01)01446-1 11833742

[38] Lees-Miller JP, Heeley DH, Smillie LB. An abundant and novel protein of 22 kDa (SM22) is widely distributed in smooth muscles. Purification from bovine aorta. Biochem J. 1987;244:705–9. 10.1042/bj2440705 PMC1148053 3446186

[39] Régent A, Ly KH, Lofek S, Clary G, Tamby M, Tamas N, et al. Proteomic analysis of vascular smooth muscle cells in physiological condition and in pulmonary arterial hypertension: Toward contractile versus synthetic phenotypes. Proteomics. 2016;16:2637–49. 10.1002/pmic.201500006 27458111

[40] Chen S, Qin S, Pei C, Zhang S. Expression and significance of NELIN and SM22α in varicose vein tissue. Exp Ther Med. 2015;9(3):345–9. 10.3892/etm.2015.2170 PMC4316901 25667639

[41] Tetzlaff F, Fischer A. Control of blood vessel formation by notch signaling. Mol Mech Notch Signal. 2018;1066:319–8. 10.1007/978-3-319-89512-3_16 30030834

[42] Boucher J, Gridley T, Liaw L. Molecular pathways of notch signaling in vascular smooth muscle cells. Front Physiol. 2012;3:81. 10.3389/fphys.2012.00081 PMC3321637 22509166

[43] Baeten J, Lilly B. Notch signaling in vascular smooth muscle cells. Adv Pharmacol. 2017;78:351–82. 10.1016/bs.apha.2016.07.002 PMC5964982 28212801

[44] Zhang C, Li H, Guo X. FOXC2-AS1 regulates phenotypic transition, proliferation and migration of human great saphenous vein smooth muscle cells. Biol Res. 2019;52:59. 10.1186/s40659-019-0266-z PMC6894326 31801629

